# Dentistry as a Profession

**Published:** 1862-05

**Authors:** 


### DENTISTRY AS A PROFESSION.

WE commend to the careful consideration of our readers the article in the present number of the Register, from the *Medical and Surgical Reporter*, upon "Dentistry as a Profession." The views there taken are by one outside of the dental ranks, yet fully competent to judge whereof he speaks.

The positions taken by him are all correct, and will, perhaps, be admitted as such, by the great majority of dentists; yet how few even make an attempt to arrive at this standard of attainments! Very many seem to rest satisfied with a superficial knowledge of the fundamental principles upon which dental science and practice are based.

It is a matter of congratulation and rejoicing, however, that this state of things is being changed; those who will not come up to the standard can scarcely expect to receive a full professional recognition, either by dentists, physicians, nor indeed much longer by the people.

A dental studentship is no longer a short, easy road, that may be passed over in a few days or a few weeks, with the attempt to digest only one simple thought or idea.

We have oftentimes experienced feelings of exceeding regret and annoyance at the deficiency of attainments in our profession, but then we take courage when we remember that ours is not the only profession in this condition. All have the same kind of discouragements with which to contend; but we hope for better things. T.

